# Editorial: Autoimmune Blistering Diseases

**DOI:** 10.3389/fimmu.2020.01614

**Published:** 2020-07-22

**Authors:** Cezary Kowalewski, Takashi Hashimoto, Pascal Joly

**Affiliations:** ^1^Department of Immunodermatology, Medical University of Warsaw, Warsaw, Poland; ^2^Department of Dermatology, Osaka City University Graduate School of Medicine, Osaka, Japan; ^3^Deparment of Dermatology, Rouen University Hospital, Center de référence des maladies bulleuses auto-immunes, Normandie University, Rouen, France

**Keywords:** pemphigus, pemphigoid, dermatitis herpetiformis, pathgenesis of autoimmune blistering diseases, treatment of autoimmune blistering diseases

Autoimmune blistering skin diseases are an extremely fascinating field of research. In recent years, most of the antigens recognized by autoantibodies in pemphigus and pemphigoid diseases have been characterized, and the pathomechanism of these diseases has been largely understood. However, several issues still need to be clarified. The content of this Research Topic consists of a series of original researches, review articles, and case reports, which expand our knowledge for the pathogenesis and new treatment strategies.

Di Lullo et al. identified a novel target antigen in pemphigus vulgaris (PV). Applying a method of the efficient immortalization of IgG+ memory B cells, the authors isolated human monoclonal antibody reactive with a non-Dsg antigen. Immunoprecipitation and immunoblotting studies on keratinocyte extracts identified α-catenin as the putative antigen, which was further confirmed by immunoblotting on the recombinant protein. The role of anti-α-catenin antibody in the mechanism of blister formation in pemphigus should be further analyzed.

In contrast to anti-α-catenin antibody, the pathogenic role of autoantibodies to desmogleins (Dsgs) and desmocollins (Dscs), desmosomal cadherins, are well-established. However, the precise mechanism of acantholysis is only partly understood. It is known that binding of pemphigus autoantibodies to Dsg3 leads to gross morphological changes in keratinocytes due to internalization of desmosomal proteins and keratin retraction. These structural alterations induced by pemphigus antibodies are mediated by intracellular signaling events via various molecules, such as p38 mitogen-activated protein kinase (p38MAPK) and extracellular-signal regulated kinase (ERK). Signaling mechanisms increase cytokine secretion and phosphorylation of structural proteins, which eventually lead to the loss of cell adhesion. It has been reported that single nucleotide polymorphism in the promoter region of the gene encoding suppression of tumorogenicity 18 (ST18) causes ST18 up-regulation, increase in cytokine secretion and more prominent loss of keratinocyte cohesion induced by pemphigus antibodies. Radeva et al. studied the effects of pemphigus anti-Dsg3 antibodies on cytokine secretion and ERK activity in human keratinocytes transfected by ST18 construct. Without ST18 overexpression, both human and mouse pemphigus antibodies induced loss of keratinocyte cohesion and fragmentation of Dsg3 along cell borders, but the release of pro-inflammatory cytokines was not altered significantly. This result indicates that cytokine expression is not necessarily required for loss of keratinocyte cohesion in this disease model. Upon ST18 overexpression, fragmentation of cell monolayers increased significantly as well as production of IL-1α and IL-6. These results partially explain ethnic susceptibility and familial occurrence of PV described in the previous reports, and provide an evidence for a genetic predisposition to PV.

Oktarina et al. studied the fate of desmosomal proteins in the biopsies taken from perilesional skin of pemphigus foliaceus (PF) patients by double immunofluorescence staining method using battery of antibodies directed to different epitopes of the desmosomal proteins, Dsg1, Dsg3, Dsc1, Dsc3, plakoglobin (PG), desmoplakin, and plakophilin 3, as well as markers of lysosomes and endosomes. This study showed that staining for either cathepsin D or LAMP-1 did not overlap with Dsg1 or PG, suggesting that lysosomes have no role in the clearing process. The co-localization of Dgs1, plakoglobin and early endosomal antigen 1 suggested that endocytosis is part of the pathogenic process in PF.

French Bullous Disease Research Group for the first time showed that rituximab was more effective than a standard oral corticosteroid treatment in pemphigus, and that Dsg-specific-B cells (Dsg-positive B cells) were still detectable during the B cell recovery even in patients in clinical remission. In the article by Hébert et al. the authors characterized Dsg-positive B cells in patients in clinical remission after rituximab or corticosteroid treatment in comparison to those at baselines in the patients' active stages. They studied the expression profiles of 31 genes related to inflammatory cytokines, TNF receptors, and activation markers. At baseline, the patients' autoreactive B cells showed a significantly higher expression of genes encoding pro-inflammatory cytokines than non-autoreactive B cells. The gene expression profiles of Dsg-positive B cells collected from patients in clinical remission after rituximab treatment were similar to those of Dsg-positive B cells at baseline, except for lower expressions of the IL-1β and the CD27 memory marker genes. This study showed that the gene expression profiles in Dsg-positive autoreactive B cells are different from those in non-autoreactive B cells, and that rituximab and corticosteroids have different effects on the gene expression in autoreactive Dsg-positive B cells in pemphigus patients.

In addition to the above-mentioned original researches on the pathogenesis of pemphigus, there are two interesting case reports by Schauer et al. on radiation-associated pemphigus and by Solimani et al. on thymoma-associated paraneoplastic pemphigus.

In the review article by Didona et al., the authors presented current state of knowledge on etiopathogenesis, diagnostics, and therapeutic strategies in mild and severe form of pemphigus. Authors analyzed efficacy of corticosteroids, several immunosuppressants, intravenous immunoglobulin (IVIg), immunoadsorption and rituximab, and proposed algorithms for the induction and maintenance therapies, and therapy for relapse. In this review, the authors also presented future perspectives of pemphigus management, including the promising treatment approach using chimeric autoantibody receptor (CAAR)-T cell.

In the review by Izumi et al., the authors presented several promising ongoing clinical trials on pemphigus. These include rituximab, a novel defucosylated human IgG1 monoclonal antibody to BAFF receptor (VAY736) which depletes B cells, monoclonal antibody to neonatal Fc receptor which reduces circulating IgG antibody level, a Bruton's tyrosine kinase inhibitor (RN1008) which inhibits B cell receptor signaling and reduces B cell activation in autoimmunity, and polyclonal regulatory T cells (PolyTregs). The authors also summarized the ongoing clinical trials on bullous pemphigoid (BP) patients resistant to standard therapies, including IVIg, rituximab, a recombinant fully humanized monoclonal antibody targeting IL-17 (ixekizumab), a fully human monoclonal antibody targeting eotaxin-1 (bertilimumab) which impairs eosinophil infiltration into the skin.

The role of IL-17 and IL-23 for neutrophilic and eosinophilic infiltrations in the BP relapse was studied by Giusti et al..

A Japanese group previously reported that autoantibodies in BP induced by dipeptidyl peptidase-IV inhibitors (DPP4i), a widely used antihyperglycemic drugs, tend to target epitopes on non-NC16A regions of BP180. In the article by Mai et al. of the same group, the authors examined BP180 epitopes using various domain-specific recombinant proteins and plasmin-digested recombinant BP180 for 18 sera of DPP4i-induced BP targeting the non-NC16A domain. They showed that IgG1-class autoantibodies targeting epitopes on the processed extracellular domain of BP180, i.e., LABD97.

In the article by Ujiie et al. of the same group, the authors studied the humoral immune response to intra- and extracellular epitopes of BP180 using an active BP mouse model, in which BP is induced by the adoptive transfer of spleen cells from wild-type mice immunized with human BP180-expressing skin grafting to immunodeficient BP180-humanized (Rag-2^−/−^, mouse Col17^−/−^, human COL17^+^) mice. Authors found that the immune response to the extracellular domain epitopes of human BP180, particularly the NC1*6A* domain, triggered intramolecular epitope spreading to intercellular epitopes of human BP180 and intermolecular epitope spreading to murine BP230.

Tie et al. studied the mechanism of blister formation in BP using keratinocyte culture stimulated with IgG anti-BP180 antibodies in patient sera. Morphological and functional changes were evaluated by electron microscopy and a battery of laboratory methods. The authors found alterations in the cell membrane structure and accumulation of intracellular vesicles. These morphological changes in the treated cells were accompanied by dysfunctional mitochondria, increased production of reactive oxygen species, increased motility, and cell detachment. These cellular alterations are reversed by pharmacological inhibitors of Rac1 or proteasome pathway molecule, suggesting that Rac1 and proteasome activation are involved in the effects of BP antibodies on cultured keratinocytes.

We hope that this Research Topic on autoimmune blistering skin diseases will be of interest to the scientific community. The articles published in this issue should expand our knowledge about the pathogenesis of these diseases, leading to development of new effective treatments.

## Author Contributions

All authors listed have made a substantial, direct and intellectual contribution to the work, and approved it for publication.

## Conflict of Interest

The authors declare that the research was conducted in the absence of any commercial or financial relationships that could be construed as a potential conflict of interest.

